# Synthesis of CdSe Quantum Dots in Two Solvents of Different Boiling Points for Polymer Optical Fiber Technology

**DOI:** 10.3390/ma17010227

**Published:** 2023-12-31

**Authors:** Anna Kiczor, Paweł Mergo

**Affiliations:** Laboratory of Optical Fibers Technology, Institute of Chemical Sciences, Faculty of Chemistry, Maria Curie-Skłodowska University in Lublin, M. Curie-Skłodowska Sq.5, 20-031 Lublin, Poland

**Keywords:** quantum dots, PMMA, polymer, CdSe, optical fiber, POF

## Abstract

Polymer materials find many applications in various industries. Efforts are being made to obtain structures with increasingly better properties. It is necessary not only to obtain new materials but also to modify existing structures. Such is the situation with polymer optical fibers. The widespread use of polymer optical fibers is impossible, due to their very high optical losses compared to glass optical fibers. The solution to this problem can be the manufacturing of polymer active optical fibers. Active fibers are the basic components of fiber optic amplifiers and lasers that allow the direct amplification of light inside the fiber. In order for their operation to be the most effective, it is necessary to use dopants. The most commonly used are lanthanide ions isolated from the polymer network, active organic dyes, and quantum dots. These dopants are characterized by very high luminescence and long glow times. Quantum dots of CdSe are made using two organic solvents that differ in boiling points—hexane (a low-boiling solvent with a boiling point of 69 °C) and 1-octadecene (a high-boiling solvent with a boiling point of 315 °C). This work aims to test whether the type of solvent used to obtain quantum dots affects the doping capabilities of polymer structures, from which optical fibers can then be drawn.

## 1. Introduction

Polymer optical fibers, unlike silica glass fibers, have a larger core diameter. This has a favorable effect on the splicing and coupling of optical fibers. However, they are characterized by increased scattering due to them having higher attenuation values than standard glass optical fibers. Doping them with lanthanide compounds [1,2,3,4], organic dyes [5,6] or quantum dots is possible due to low synthesis and thermo-processing temperatures and the possibility of doping using various methods. Combining a polymer matrix with the addition of active dopants makes it possible to obtain active polymer optical fibers (POFs). The addition of dopants affects numerous properties of the resulting materials, including luminescence, transmission, and functional properties. A major advantage of such polymer optical fibers is their high flexibility, low manufacturing costs, and high numerical aperture value. The polymers mainly used in optical fiber technologies are polymethylmethacrylate (PMMA), polycarbonate (PC), and polystyrene (PS). POFs are mainly made from polymethylmethacrylate (PMMA). It is characterized by very good UV resistance, near-UV and VIS transmission, and a visible light transmittance of 90–92%. Due to its very high biocompatibility, this material can be used in medical applications [7,8].

Research on the effects of lanthanide addition to PMMA has been carried out for more than a dozen years. This is a very difficult task, due to the insolubility of lanthanide salts in organic solvents. The team of R. Piramidowicz [9,10,11] studied the properties of such structures in a different spectral range and by adding lanthanides under different forms to methyl methacrylate. The study in [12] compared the UV luminescence properties of YF_3_, LaAlO_3_, Al_2_O_3_, and Y_2_O_3_ compounds subsidized with Pr^3+^ ions, which were then added to PMMA-based composites. Work on combining the PMMA matrix with organic dyes is also progressing rapidly [13,14]. The study in [15] tested the possibility of using PMMA doped with organic dyes as a dye laser.

Quantum dots (QDs) are semiconductor nanocrystals with properties that are intermediate between semiconductors and quantum particles in size at 2–10 nm. By modifying the composition and size, luminescence can be obtained over the full range of the spectrum, that is, from UV to IR. The smaller the size of the nanoparticle, the more the color of the emitted light is shifted toward blue, while the larger it is, the more it is shifted toward red [16,17,18]. There are many methods for obtaining quantum dots. The smallest structures can be obtained using the colloidal method, during which semiconductor crystals are precipitated out of a solution. The difficult part of this method is preventing the nanoparticles from clumping together. It is necessary to use a suitable organic solvent to prevent aggregation and the uncontrolled growth of the structure. The resulting quantum dots are covered with a homogeneous, tight envelope, which further protects them from the external environment. Typical stabilizing ligands include trioctylphosphine oxide (TOPO), carboxylates, amines, and phosphines. TOPO (trioctylphosphine oxide) is often used as a coordinating ligand and solvent in QD synthesis. It binds to the surface of QDs and stabilizes them, forming a protective layer. The concentration of TOPO can affect the size and shape of the QDs. Higher concentrations of TOPO can lead to larger particle sizes. It also provides effective surface passivation, reducing surface defects and improving the optical properties of QDs. Carboxylates, such as oleic acid, are commonly used ligands for QDs. They can replace TOPO or be used in combination with it. They can affect the size and surface charge of QDs and the dispersibility of QDs in different solvents. Carboxylate groups provide functional handles for further modification and bioconjugation, making QDs suitable for biomedical applications. Amines and phosphines can be used as alternative ligands to TOPO or in combination with other ligands to control the surface chemistry of QDs. They can contribute to surface passivation and increase QD stability. The choice of stabilizing ligands can affect the energy levels of QDs, affecting their optical properties, such as absorption and emission spectra. Ligands that provide effective surface passivation contribute to brighter and more stable QDs with reduced, non-radiative recombination. The nature of stabilizing ligands affects the colloidal stability of QDs. Well-designed ligands prevent aggregation and maintain colloidal stability over time. Ligands that provide effective passivation help prevent the exposure of the QD surface to environmental factors, reducing degradation. The choice of stabilizing capping ligands during the synthesis of colloidal semiconductor quantum dots is a critical factor in determining their physical and physicochemical properties. It affects size, shape, surface chemistry, stability, and optical properties, making it essential to tailor the ligands based on the desired QD application. The solvent, 1-octadecene (ODE), as a non-coordinating solvent requires oleic acid, which acts as a ligand that binds to the surface. The big advantages of 1-octadecene are that it has a high boiling point (315 °C), it is a liquid at room temperature, and the kinetics of the particle formation can be easily controlled [19,20,21,22,23].

The use of a high-boiling organic solvent in the synthesis of colloidal CdSe quantum dots, especially when they are intended for polymer doping, serves several important purposes. Chief among these is the ability to control temperature. These solvents have elevated boiling points, allowing for higher reaction temperatures during quantum dot synthesis. This can be crucial in controlling the nucleation and growth of quantum dots, affecting their size, size distribution, and crystallinity. Temperature is a critical parameter in the synthesis process, and high-boiling solvents provide a stable environment for controlled reactions. Another important aspect is the solubility of the precursor ions needed to obtain quantum dots. Some precursors used in quantum dot synthesis may have limited solubility in solvents with lower boiling points. Solvents with high boiling points can dissolve a wider range of precursors, ensuring that all the necessary components are well mixed and available for quantum dot formation. Synthesis of CdSe quantum dots involves high-temperature reactions, and high-boiling solvents can help prevent the thermal decomposition of precursors or formed quantum dots. This is important for preserving the integrity of the quantum dots and for avoiding unwanted byproducts that can affect their optical and electronic properties. The kinetics of the reaction must also be kept in mind. The use of high-boiling solvents can affect reaction kinetics, allowing for the slower and more controlled nucleation and growth of quantum dots. This can help to unify the size and shape of quantum dots, which is crucial for their optical properties. It is also worth considering that solvents with high boiling points are often more compatible with polymer matrices. Once quantum dots are synthesized, these solvents can be retained in the polymer matrix during the doping process. This retention can facilitate the uniform distribution of quantum dots in the polymer, improving the homogeneity of the composite material. It should be noted that the choice of solvent depends on the specific requirements of the synthesis process, the nature of the quantum dots, and compatibility with the polymer matrix. The goal is to achieve a well-controlled and reproducible synthesis of colloidal quantum dots and to facilitate their efficient integration into the polymer to achieve the desired optical and electronic properties.

Researchers have been trying for years to combine quantum dots with polymers to improve stability and thermal and mechanical properties. Semiconductor quantum dots have unique electronic and optical properties, due to their size and quantum confinement effects. At the beginning of the 21st century, scientists actively explored various semiconductor materials, including those in Group II (alkaline earth metals), Group I (alkali metals), and Group IV (main group elements) in the periodic table, to create quantum dots with tailored properties [24,25,26,27,28]. Key features and advances during this period may include synthesis methods. Scientists developed novel synthesis methods to precisely control the size, shape, and composition of quantum dots. These methods often included colloidal chemistry, chemical vapor deposition, or other techniques to engineer quantum dots with specific properties. In addition, the ability to tune the optical properties of quantum dots became a major goal to be pursued. Quantum dots of different sizes exhibit different band gaps, allowing scientists to control the colors of light emitted or absorbed by these materials. Such tunability is crucial for optoelectronics applications, such as light-emitting diodes (LEDs) and quantum dot displays. In the early 21st century, interest in the potential applications of semiconductor quantum dots in electronics also grew. Quantum dots have been studied for their use in transistors, memory devices, and other electronic components because of their unique electronic properties [29,30,31].

In a paper [32], CsPbBr_2_ quantum dots were combined with a PMMA matrix, and an increase in structure transparency was noted. Alicia De San Luis and her team [33] created hybrid cross-linked PS/QD/PMMA nanofibers, which had good optical stability, due to the presence of CdSe/ZnS quantum dots. They have been used with positive results in volatile organic compound (VOC) sensors as a luminescent material. Only recently, researchers have successfully created a thin film of PMMA doped with carbon quantum dots [34]. It was used in solar cells, and down-conversion was made possible by using carbon QDs. The thin PMMA film was directed with the active part toward the illumination. Analysis of the device’s performance showed that the use of such a film resulted in an improved photocurrent and fill factor and consequently increased the device’s efficiency. It is also worth noting the study of Saidzhonova et al. [35], who studied the effect of adding cadmium halide (NPL CdSe) to a PMMA polymer matrix. Cadmium halide does not, like CdSe quantum dots, exhibit the continuous tunings of absorption and emission, i.e., it has a limited range of applications. However, the researchers demonstrated many positive features of such a solution, carefully studied the effect of the thickness of the active layer, and checked the optoelectronic properties. They showed that the addition of NPL causes a shift of emission peaks toward the red color. There are also studies in which CdSe/ZnS quantum dots made with toluene were added to PMMA at the prepolymerization stage [36]. Bueno et al. [37] fabricated a temperature sensor based on CdSe and CdTe quantum dots embedded in a PMMA matrix. They fabricated planar waveguides. They showed that when the samples were heated from 25 to 50 °C, the QD waveguide photoluminescence showed a strong decrease in intensity, with a quadratic dependence of temperature. They obtained a temperature resolution of 0.1 °C. Abuzaida et al. [38] fabricated a PMMA matrix doped with CdSe/ZnS QDs. They modified it using MPTMS and checked how the surface properties of such a structure changed. In the article [39], CdSe quantum dots of three different sizes were made using a colloidal method. They doped the PMMA with the obtained quantum dots in different ratios. Such quantum dot-doped polymer matrices were deposited on LEDs to study the effects of different weight ratios of CdSe QDs and PMMA on the performance of white LEDs. Other teams of researchers [40,41,42,43,44,45,46] have also been involved in creating composites and structures combining polymers with quantum dots. These were mainly films and thin films with the addition of different types of quantum dots, depending on further applications.

In this work, unlike others, we aimed to make CdSe quantum dots in two different organic solvents—hexane (with a boiling point of 69 °C) and 1-octadecene (with a boiling point of 315 °C). The properties of the two quantum dot solutions were compared and then added to the polymerization mixture. This work aims to test whether the type of solvent used to obtain quantum dots affects the possibility of obtaining doped polymer structures, from which polymer optical fibers can then be extracted. Table 1 shows a comparison of the organic solvents used.

## 2. Materials and Methods

Materials: 1-octadecene (ODE for synthesis, SIGMA-ALDRICH, Darmstadt, Germany), cadmium oxide (CdO, ≥99.99% trace metals basis, SIGMA-ALDRICH, Germany), trioctylphosphine (TOP, 97%, SIGMA-ALDRICH, Germany), selenium powder (Se, -100 mesh, 99.99% trace metals basis, SIGMA-ALDRICH, Germany), oleic acid (≥99%, ROTH), hexane (ReagentPlus, ≥99%, SIGMA-ALDRICH, Germany), thioglycolic acid TGA (98%, SIGMA-ALDRICH, Germany), benzoyl peroxide (for synthesis, SIGMA-ALDRICH, Germany), and methyl methacrylate (MMA, 99%, SIGMA-ALDRICH, Germany).

**Figure 1:**
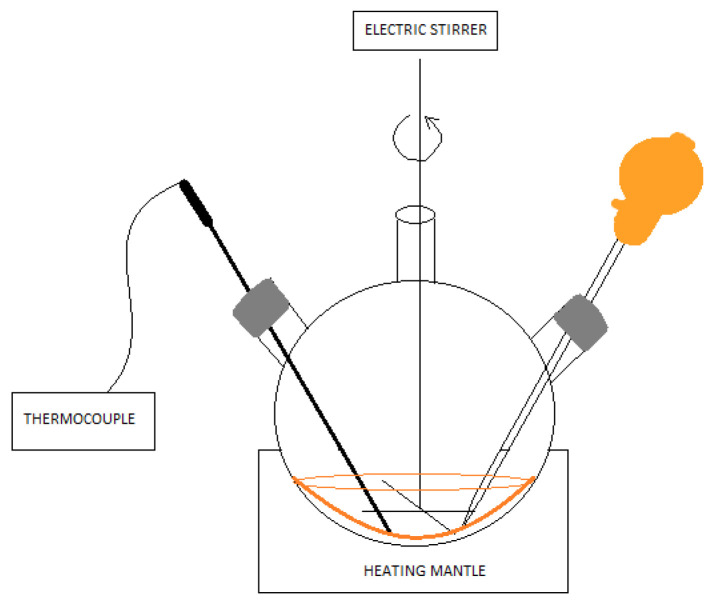
Schematic of CdSe QD synthesis using the colloidal method.

The synthesis was performed similarly to [22], but an electric stirrer was used to thoroughly mix the substrates. In addition, the injection temperature of the selenium ion precursor was reduced. The solution containing the selenium ion precursor was made in a flat-bottomed flask. Amounts of 0.03 g of powdered selenium (Se, -100 mesh, 99.99% trace metals basis, SIGMA-ALDRICH, Germany), 0.4 cm^3^ trioctylphosphine (TOP, 97%, SIGMA-ALDRICH, Germany), and 5 cm^3^ 1-octadecene (ODE for synthesis, SIGMA-ALDRICH, Germany) were added. The whole mixture was mixed thoroughly, and a colorless TOPSe solution was obtained. To a three-neck round-bottom flask was added 0.013 g of cadmium oxide (CdO, ≥99.99% trace metals basis, SIGMA_ALDRICH, Germany), 0.6 cm^3^ of oleic acid (≥99%, ROTH), and 5 cm^3^ of 1-octadecene. The whole mixture was placed in a heating bowl and heated until the cadmium oxide was completely dissolved at about 180 °C. The system for performing the synthesis additionally included a thermocouple for continuous temperature control and a magnetic stirrer (Figure 1). After this time, 1 mL of the previously prepared solution containing selenium ions was added. Immediately, there was a gradual change in color from colorless to yellow, orange, and red until it became maroon. Samples were taken during the process after 60, 120, 300, 600, 1800, and 3600 s. A sample taken after 300 s was used for further studies. Similarly, the synthesis was performed using hexane (ReagentPlus, ≥99%, SIGMA-ALDRICH, Germany) as an organic solvent. However, it had to be remembered that the boiling point of hexane is 69 °C, so the reaction was carried out at much lower temperatures. As much of the low-boiling solvent as possible was evaporated so that it would not cause imperfections in the subsequent processing of the polymer. A sample taken after 300 s was also used for later studies.

For the results obtained, it was possible to calculate the double-band conversion. The value of the double-band conversion indicates the amount of the reacted monomer, which is of crucial importance, since it affects the quality class and thermal stability of the polymer. The double-band conversion was calculated according to the equation:

$$\pi =[1-(\frac{A1720\;monomer}{A810\;monomer}\times \left(\frac{A810\;polymer}{A1720\;polymer}\right)]\times 100\%$$

where: 

A_1720 monomer_ is the band absorbance at the wavelength 1720 cm^−1^ derived from the monomer,

A_810 monomer_ is the band absorbance at the wavelength 810 cm^−1^ derived from the monomer,

A_810 polymer_ is the band absorbance at the wavelength 810 cm^−1^ derived from the polymer,

A_1720 polymer_ is the band absorbance at the wavelength 1720 cm^−1^ derived from the polymer, and

Π is the double bond conversion [47].

## 3. Results and Discussion

**Figure 2:**
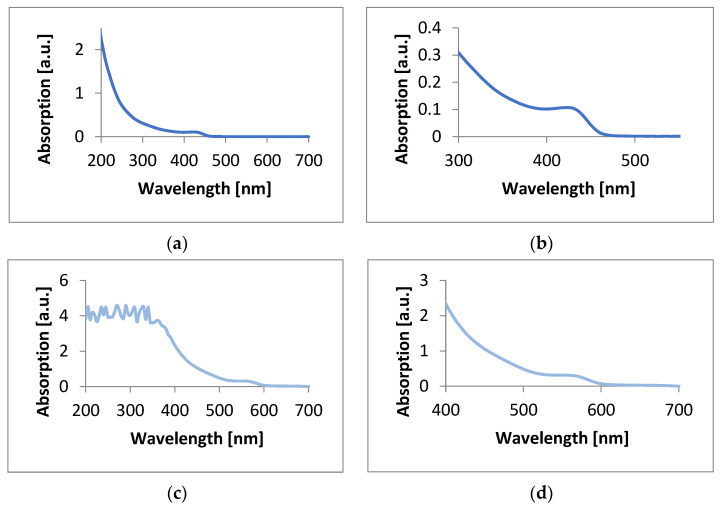
Absorption plots of CdSe quantum dots prepared using (**a**) hexane, (**b**) hexane with an enlarged fragment containing the absorption peak, (**c**) 1-octadecene, and (**d**) 1-octadecene with an enlarged fragment containing the absorption peak.

Absorption and emission spectra of CdSe quantum dot solutions were performed using a Hitachi F-7000 Fluorescence spectrophotometer (Hitachi High-Tech Corporation, Tokio, Japan) and an Agilent Cary 60 UV-VIS spectrophotometer (Agilent Technologies, Santa Clara, CA, USA). The spectrum of CdSe QDs made using hexane as an organic solvent showed a small peak appearing at 435 nm, corresponding to the blue color. Quantum dots made using 1-octadecene showed a clear peak at 575 nm, corresponding to the red color (Figure 2).

**Figure 3:**
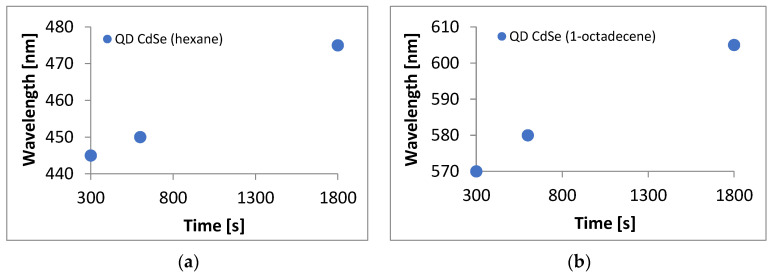
Graphs of the dependence of sampling time on the absorption maximum peak for quantum dot solutions prepared using (**a**) hexane, (**b**) 1-octadecene.

Graphs of the dependence of the sampling time on the absorption peak maximum were made for quantum dot solutions made from both solvents (Figure 3). UV-VIS wavelength reading error was ± 0.08 nm, and the error was related to the sampling time of 2 s. In the case of CdSe quantum dots made with hexane, the function for the formula was y = 0.0202x + 438.45. In the case of CdSe quantum dots made with 1-octadecene, the function had the formula y = 0.0226x + 564.64. The directional coefficients of both graphs were very similar, confirming that similar structures were obtained. It can be seen that the relationships in both cases were practically linear and homogeneous. This allows us to conclude that in both cases, we obtained the expected QD CdSe structures.

**Figure 4:**
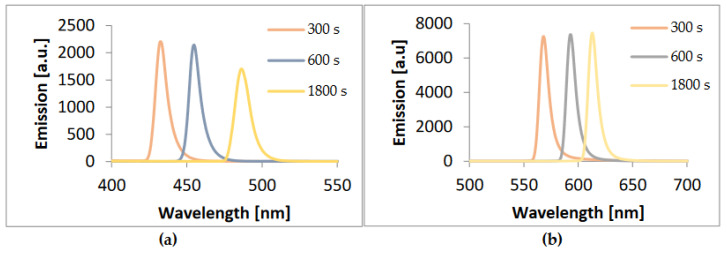
Emission plots for three CdSe QD samples obtained after different synthesis times with (**a**) hexane (**b**) 1-octadecene.

Emission spectra were performed for three samples taken at different times after the selenium ion precursor was injected, i.e., after 300, 600, and 1800 s. Figure 4 shows the emission spectra of the obtained structures. A multiplier of 5 was applied to both spectra. In both cases, it can be seen that the longer the synthesis lasted, the more the emission peak maximum shifted towards longer wavelengths. The peaks were narrow and well-defined. The spectra of quantum dots produced using 1-octadecene were characterized by very strong luminescence, which resulted in spectral oversaturation. In the case of quantum dots obtained using ODE, the peaks were more intense, characterized by very high luminescence, which affected the slight oversaturation of the samples. The spectra were taken using the same parameters in order to reliably compare emission intensities.

The full width at half maximum (FWHM) is a measure of the width of a peak in a spectrum at half of its maximum intensity. In the context of emission spectra, it provides information about the range of wavelengths over which the emitted light is distributed. In this case, we had three emission spectra of samples of the same compound, taken at different synthesis times. For the CdSe quantum dots made with hexane, their FWHM readings were 9 nm, 9.2 nm, and 10.2 nm. For the CdSe QD samples made with 1-octadecene, they were 9.6 nm, 11 nm, and 11 nm, respectively. The different FWHM values suggest that the emission profiles of the three samples are not identical. Each sample emits light in a slightly different wavelength range. A smaller FWHM suggests narrower peaks, while a larger FWHM suggests wider peaks. Changes in FWHM can indicate differences in environmental conditions, that is, even a small change in temperature can affect emission characteristics.

Using thermal free-radical polymerization, pure PMMA and PMMA/admixture were produced. Benzoyl peroxide (for synthesis, SIGMA-ALDRICH, Germany) was used as the initiator, and thioglycolic acid TGA (98%, Aldrich) was used as the chain transfer agent [39,40,41]. CdSe quantum dots obtained earlier were added to the polymerization mixture, which also contained methyl methacrylate and polymerized. Thermal polymerization was carried out in 14 mm diameter glass tubes. Pure PMMA, PMMA with CdSe quantum dots produced using 1-octadecene, and PMMA with quantum dots produced using hexane were obtained. The resulting structures were trimmed into cylinders of equal length, polished, and further tested [48,49,50].

**Figure 5:**
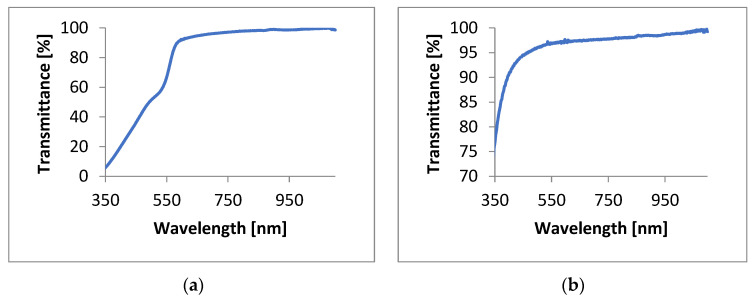
Graph of the PMMA/CdSe QD transmittance generated with the use of (**a**) 1-octadecene and (**b**) hexane.

Transmittance spectra were obtained at room temperature using a single beam visible spectrophotometer V-5600PC (bandwidth, 2 nm; wavelength accuracy, ±0.8 nm; wavelength repeatability, 0.3 nm; stability, ± 0.002 A/h, and stability ± 0.002 A/h). The transmittance was measured for wavelengths ranging from 320–1100 nm (Figure 5).

**Figure 6:**
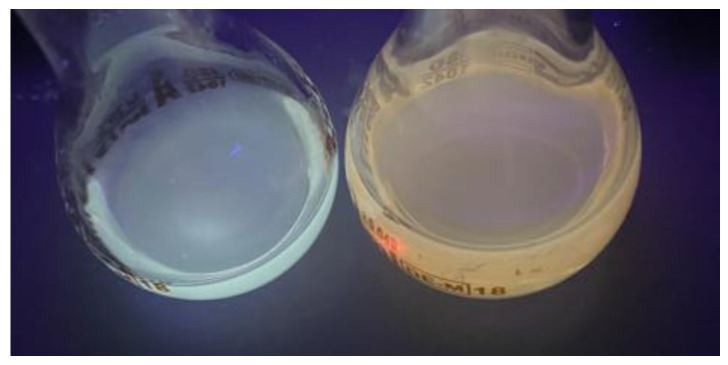
CdSe quantum dot solutions made with hexane (blue solution) and 1-octadecene (orange solution).

Figure 6 shows the solutions of CdSe quantum dots in both solvents using a UV lamp. The solution of CdSe QDs produced using hexane showed a blue color after UV lamp exposure, while the solution of CdSe QDs using 1-octadecene showed a dark orange color. They corresponded to 2 nm and 5 nm sizes, respectively. This was reflected in the emission spectra performed.

**Figure 7:**
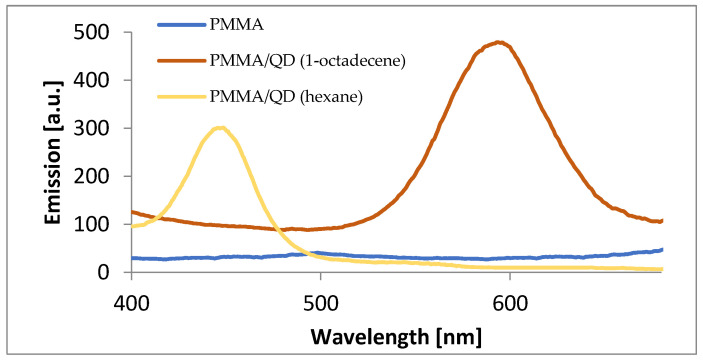
Comparison of the emission spectra of PMMA samples (blue line), PMMA with QDs made with hexane (yellow line), and PMMA with QDs made with 1-octadecene (brown line).

Photoluminescence spectra of pure PMMA, PMMA with the addition of quantum dots made with hexane, and PMMA with the addition of quantum dots made with 1-octadecene were performed (Figure 7). It can be seen that the emission peaks for PMMA samples with quantum dots corresponded respectively with the emission peaks of the solutions of CdSe quantum dots alone. This shows that the CdSe quantum dots encapsulated in the polymer structure still exhibited very good luminescence properties. Well-defined emission peaks with a symmetrical shape were obtained. The sample of pure PMMA was practically linear.

**Figure 8:**
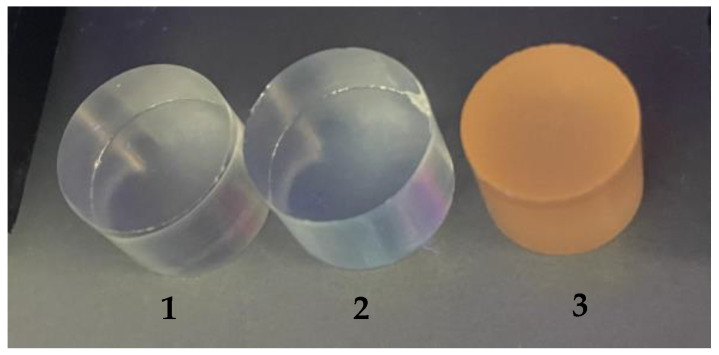
Luminescence of the obtained samples: 1—pure PMMA, 2—PMMA with the addition of CdSe quantum dots made using hexane, and 3—PMMA with the addition of CdSe quantum dots made using 1-octadecene.

Emission was also confirmed using a UV lamp (Figure 8). Number one denotes a sample of pure PMMA, number two denotes a sample of PMMA containing CdSe quantum dots made using hexane, and number three denotes a sample of PMMA containing CdSe quantum dots made using 1-octadecene as an organic solvent. The number 1 sample showed no change in coloration; the sample was still colorless. Sample number 2 showed a blue color, and sample number 3 showed an orange–red color, which may actually correspond to the emissions at the wavelengths shown in Figure 7.

**Figure 9:**
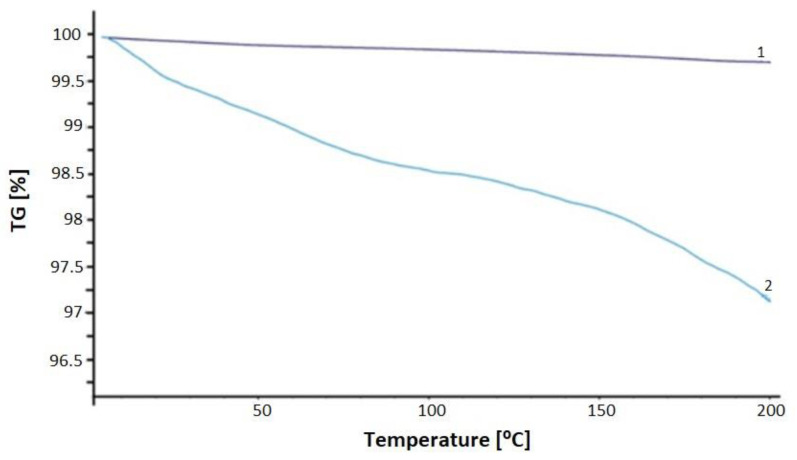
TG plot of PMMA/QD CdSe made in 1-octadecene [1-purple line]; TG plot of PMMA/QD CdSe made in hexane [2-blue line].

Termogravity (TG) analyses of the obtained structures were performed using STA 449 F1 Jupiter NETZSCH (NETZSCH, Selb, Germany) at a heating rate of 10 °C/min in the temperature range of 0–200 °C. The mass of the sample was about 10 mg. A type-S TG-DCS sensor thermocouple was used. An empty Al_2_O_3_ crucible was used as a reference. TG for the sample containing pure PMMA is described in [51]. Sample No. 1 determined a sample made of PMMA doped with CdSe QD using 1-octadecene as a high-boiling organic solvent. A very small percentage decrease in the weight of the sample can be seen. The graph was practically straight, with a decrease of 0.3%. This is an excellent result, confirming that this sample is thermally stable and a good material for further use as a polymer optical fiber. Sample No. 2 showed a percentage decrease in the weight of the sample as the temperature increased. At 200 °C, it was almost 3%. This was not a large decrease, but was is enough to reject this sample as a material for further fiber optic processing (Figure 9).

**Figure 10:**
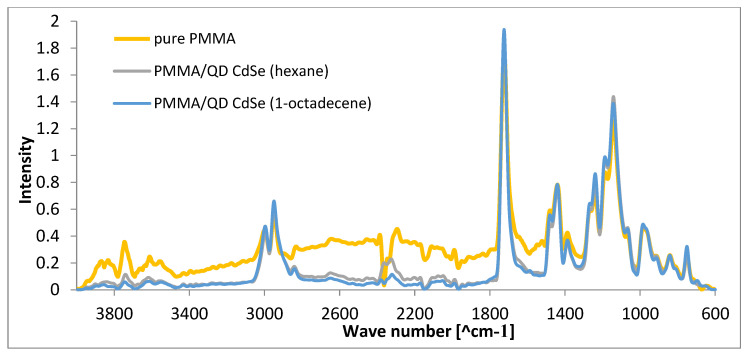
ATR/FT-IR spectra of pure PMMA, PMMA/QD CdSe (hexane), and PMMA/QD CdSe (1-octadecene).

Figure 10 shows ATR-FTIR spectra recorded using a Nicolet 6700 spectrophotometer equipped with a Diamond Smart iTR accessory in the 4000–600 cm^−1^ range. During ATR-FT-IR analysis, analyses of each of the 128 scans were performed for all samples. All the spectra were normalized, and the band at 1720 cm^−1^ was chosen as the reference [52]. This band is related to a carbonyl group that does not change during the thermal treatment [53].

PMMA is a widely studied polymer, and its ATR/FT-IR spectrum typically shows characteristic peaks associated with specific vibrational modes. The spectrum shows several characteristic peaks from bonds found in this polymer. The carbonyl stretch at about 1720 cm^−1^ is associated with the C=O bond in the ester group. The -C-O- bond stretching (ester) occurs at 1238 cm^−1^, C-C stretching occurs at 1145 cm^−1^, and C-O-C stretching occurs at 1120 cm^−1^. These peaks correspond to the different vibrational modes of the molecular groups present in PMMA. The peaks in the 1435 cm^−1^ and 750 cm^−1^ regions are associated with CH_3_ deformation. The FT-IR spectra of CdSe quantum dots primarily show features related to the surface ligands, which passivate the quantum dots and stabilize them. Common ligands include organic molecules like trioctylphosphine oxide (TOPO), trioctylphosphine (TOP), and oleic acid, among others. The peaks from the bonds found in these structures overlap with those from PMMA. There are no additional peaks present with PMMA/QD preforms that would not be present with pure PMMA.

The double bond conversion in the polymer (PMMA) was 94.8%; in the PMMA doped with quantum dots made with hexane, it was 94.3%, and in PMMA doped with quantum dots made with 1-octadecene, it was 93.3%. The high percentage of double bond conversion indicates that the polymerization process is quite efficient under all conditions. This may be a positive aspect in terms of obtaining a high-quality polymer product. The relatively small differences in double bond conversion percentages suggest that the quantum dot doping process is precise and consistent, as the variations are within a narrow range. The results may indicate that there is potential to further optimize the synthesis and doping process of quantum dots to increase double bond conversion in PMMA [47].

## 4. Conclusions

In this work, it was shown that the resulting structures based on the PMMA matrix allowed the formation of new optical polymer fibers with luminescent properties. The effects of the addition of CdSe quantum dots in two organic solvents with different boiling points were investigated. CdSe quantum dots were made using a colloidal method. During the synthesis of quantum dots, different types of organic solvents can be used. The paper showed a comparison between the use of a high-boiling-point solvent, ODE, and a low-boiling point, highly volatile solvent, hexane. A virtually linear relationship was shown between the absorption maximum and the sampling time of quantum dot solutions made with ODE and hexane. The emission spectra showed that the samples obtained were characterized by high luminescence. The longer the synthesis lasted, the more the emission peak was shifted towards larger wavelengths. This situation can be observed in both types of samples. The emission peaks were narrow and well-characterized, which is characteristic of quantum dots. It was confirmed that the longer the synthesis, the larger the quantum dots obtained, whose emission spectra were at increasing wavelengths. TG plots showed much higher thermal stability for CdSe quantum dots made using 1-octadecene than using hexane as an organic solvent. The mass of quantum dots made with the high-boiling-point solvent decreased by about 0.3%, and the latter decreased by about 3%. This shows the high instability of samples formed using hexane. It should be noted that when treating optical fibers, the temperature can reach 200 °C, which, with such losses in the mass of the sample, can negatively affect its properties. A weight loss of up to 1% makes this material very good for pulling out polymer optical fibers. In addition, it gives us guarantees that air bubbles will not form during processing, affecting the properties and signal conduction inside the optical fiber. All of these results show that a material with high luminescence potential was obtained for applications including polymer optical fibers (POFs).

## Figures and Tables

**Table 1:** Characteristics of the organic solvents used.

Organic Solvent	Structural Pattern	Molar Mass [g/mol]	Boiling Point [°C]	Density [g/mL]
Hexane	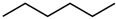	86.18	69	0.6594
1-Octadecene (ODE)	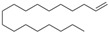	252.49	315	0.789

## Data Availability

Data are contained within the article.
